# Impact of Coronavirus Disease of 2019 Vaccine on Health and Physical Activities Among Physical Education Students in China

**DOI:** 10.3389/fpubh.2022.889311

**Published:** 2022-07-04

**Authors:** Rizwan Ahmed Laar, Zhengyi Zhang, Rashid Menhas, Lei Zhang, Shicheng Zhu, Xin Fan, Wei Wang, Shumin Li

**Affiliations:** ^1^College of Physical Education, Hubei Normal University, Huangshi, China; ^2^School of Physical Education and Sports Science, Nanjing Normal University, Nanjing, China; ^3^Research Center of Sports Social Sciences, School of Physical Education and Sports, Soochow University, Suzhou, China; ^4^School of Marxism Hubei Normal University, Huangshi, China

**Keywords:** confirmatory factor analyses, COVID-19 vaccine, physical activity performance, health, China

## Abstract

**Purpose:**

This research focused primarily on the impact of the SARS-CoV-2 Vaccine (VeroCell) on Chinese physical education (PE) students' health and physical activity (PA) performance.

**Methods:**

This study used quantitative methods and phenomenological procedures to collect and analyze data. Survey techniques were the main method used for collecting data from Chinese university students, using a self-designed questionnaire with a Cronbach's alpha α value of 0.76. To ensure the quality of the study, confirmatory factor analyses (CFA) were conducted, and the internal consistency reliability of the instrument was measured (alpha coefficient = 0.82). The determined sample size was 490 and around 90% as the minimum sample size was determined with the help of a sample size calculator. The author using factor loadings with *h*^2^ and an independent-sample *t*-test analyzed the responses of the remaining valid participants (*n* = 443 with a response rate of 90.40).

**Results:**

Most participants (around 94%) did not experience any adverse reactions that impacted their daily life activities, health, or performance during physical activity. However, about 30–40% of students felt lethargy, weakness, muscle pain, or swelling. Regarding the impact of the vaccine on daily life, there was no difference in the responses between participants who had only received one shot of the coronavirus disease 2019 (COVID-19) vaccine and those who had received two shots (*p* > 0.05 in most cases).

**Conclusion:**

The study concluded that the COVID-19 vaccine had no significant effect on PE students' daily activities, health, and PA performance. The results of this study could be used by policymakers to encourage people to get vaccinated and eradicate the isolation caused by COVID-19, which leads many people to develop various non-communicable diseases (NCDs).

## Introduction

**Figure 1:**
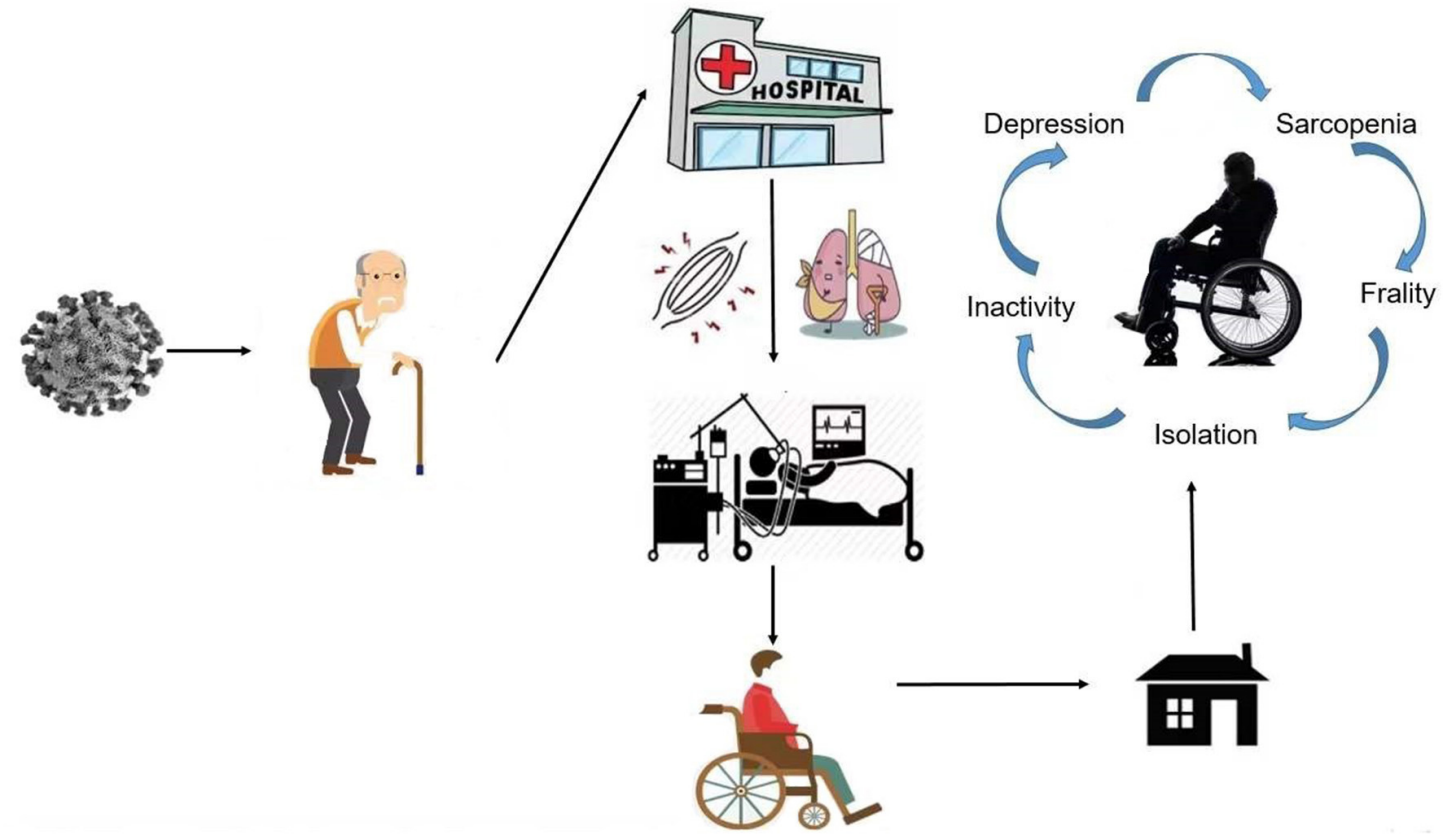
Circle of Covid-19 patient.

More than 15 million people have been victims of this pandemic, and almost 600,000 people have died from COVID-19 in nearly 210 countries worldwide. COVID-19 was first identified in Wuhan, China, in December 2019. The cause of the disease is severe acute respiratory syndrome coronavirus 2 (SARS-CoV-2) (1–3). No pandemic has taken as many lives worldwide since the influenza outbreak in Spain during World War I. Many studies have pointed out that anxiety has been the biggest problem throughout the world during the pandemic, followed by sleeping problems, and depression (4). The greatest harm posed by the virus to normal human health includes direct damage to the immune system, damage to the respiratory system, and the deterioration of underlying medical conditions, which progresses to systemic failure and death (5). Thousands of patients have been hospitalized due to COVID-19, and thousands of others were forced to quarantine. It is conceivable that this huge change in lifestyle, brought about by stagnation (confined to bed or hospitalization), isolation, and a more sedentary lifestyle, may damage the health and wellbeing of those affected and the population (6) (see Figure 1).

### Scenario of COVID-19 Vaccine Globally

As of July 2021, almost three billion doses of the COVID-19 vaccine have been administered worldwide 0.19 COVID-19 vaccines have been approved in at least one country, involving more than 11.48 billion doses. Some of these have been very effective in both clinical trials and real-world applications, and some are designed to cater to the needs of certain populations. Very few countries have approved the COVID-19 vaccine for children and adolescents (7).

Currently, almost 105 types of COVID-19 vaccine are under clinical trials, and 184 different vaccines against COVID-19 are in the pre-clinical stages (8). Most vaccines target some or all of the SARS-CoV-2 spike proteins. Various platform technologies are being studied, including viral vectors, protein subunits, RNA, DNA, and inactivation, 21, 32, 17, 10, and 16, respectively. Viral vector vaccines and messenger RNA (mRNA) nucleic acid vaccines are new formulas. They are comparatively faster to develop and manufacture, partly because developers only need to consider the genomic sequences of SARS-CoV-2 rather than physical models of viruses. One procedural limitation of mRNA vaccines is that they must be stored at ultra-low temperatures, making distribution difficult. However, there have been first phase trials of freezer stabilized mRNA vaccine (mRNA-1,283, Moderna) and lyophilized preparation (9, 10). Around nineteen COVID-19 vaccines had received regulatory approval in at least one country by June 2021 (11). Most of these had only temporary or emergency authorization, usually based on the results of temporary phase III trials.

In some cases, there are no publicly available clinical trial results. The World Health Organization (WHO) has included six unique vaccines in the emergency use list (EUL), and many other vaccines are under evaluation (12). The WHO EUL is a precondition for vaccine supply to COVID-19 Vaccine Global Access Facilities (COVAX). COVAX is an initiative of the Global Vaccine Alliance (Gavi), the Coalition of Epidemic Preparedness Innovations (CEPI), and the WHO. It is committed to accelerating the production and development of COVID-19 vaccines and ensuring equal distribution to the world via shared mechanisms.

### Vaccine Efficacy and Effectiveness

Vaccine efficacy refers to the relative risk of the same results under experimental conditions, such as observational studies (“phase IV” studies). The proportion of exact results of interest to the people who have received vaccine shot(s) is reduced (relative risk reduction) compared with unvaccinated people under “ideal” conditions, such as in phase III controlled clinical trials (13). For the COVID-19 vaccine, which is currently in use, efficacy data can only be obtained from the interim phase III trials or previous studies. According to published data, the effectiveness of the COVID-19 vaccines ranges from 50 to 95% (7). Many COVID-19 vaccines have a positive effect on severe disease, which also helps in reducing the risk of asymptomatic disease, which may decrease the spread of SARS-CoV-2. The available phase IV efficacy data are usually closely related to the results of clinical trials. This is reassuring because many countries worldwide prioritize vaccination of groups excluded or underrepresented in clinical trials, for example, the elderly and people with medical conditions, whose immune response to the vaccine may be weaker (14). The efficacy of the various COVID-19 vaccines is diverse for a range of reasons, such as inclusion criteria and definition of test results, different settings, different strains of SARS COV-2, and different infection rates in the countries where the trials were conducted. Currently, it is hard to identify the period of protection provided by the vaccines available at present (or by natural immunization). A correlative of protection (CoP) will help speed up the evaluation of new vaccines (including vaccines focusing on particular populations and variants) and the need for and timing of vaccine booster doses. The CoP has not been developed, although neutralizing antibody titers seems to be a good predictor of vaccine efficiency (15).

As the main journal of sports medicine and health globally, the Editors share a strong sense of responsibility to provide an overview of the impact of the COVID-19 vaccine on the health and performance in physical activity (PA) of physical education (PE) students. Thus, this study aims to highlight whether the COVID-19 vaccine impacts the daily life activity of the participants, mainly addressing the impact of the vaccine on the health and physical activity performance of PE students. Although there are a few studies from this perspective, regarding the side effects of COVID-19, they are, by no means, exhaustive. In addition, the existing literature (including that concerning the influenza vaccine) may oversimplify the COVID-19 vaccine's impact on the health and physical activity performance of the physical education university students. Our study has the potential to provide a fresh understanding of the relationship between the SARS-CoV-2 Vaccine (VeroCell, which is adjuvanted with aluminum hydroxide) (16) doses and the health and physical activity performance of PE students, in this case, the university students. Notably, the health status of the participants can be observed within a short time after vaccination, for impact on sports participation, long-time observation is required, and in this case, both are observed. Firstly, we have provided a summary of the pathology of COVID-19 and a brief overview of the development and efficacy of the COVID-19 vaccine. Next, we present our study methodology, and, finally, we set out the results and conclusion of the study.

## Materials and Methods

This study uses a quantitative approach, together with phenomenological measures, to collect and analyze the data (17). The main methodology of the study was survey techniques to collect data from university students in China, using a self-designed questionnaire (18–21).

### Study Participants

Like previous studies, in the first stage, the authors selected the Hubei Normal University (HBNU) based on the convenience and purposive sampling method (22, 23). It was difficult for an author to enter other universities, especially, at the time of the pandemic (further mentioned below). A total of 490 students of HBNU were contacted *via* random selection from personal links of the author and the snowball sampling technique (20, 24). After the data were collected and analyzed using Amos, 443 (242 male and 201 female) valid responses remained (*n* = 443) (Table 1). At the initial stage, we contacted the students randomly *via* our contacts and links. These students then contacted other participants using the snowball sampling approach. To avoid the different biases, this study was conducted face-to-face (25). Notably, China still maintains a policy of dynamic zero clearance, and the whole nation follows the Standard Operating Procedures (SOPs) of COVID-19. In particular, students in educational institutions are less likely to be infected with COVID-19. Taking HBNU as an example, outsiders are not normally allowed to enter schools, and students are generally not allowed to leave the school unless they comply with the school's requirements, such as health code, travel code, and 48-h nucleic acid test certificates (these policies vary from school to school and city to city, depending on the COVID-19 infection circumstances). Even national holidays are postponed (again, not by all educational institutions, the policies vary according to the COVID-19 situation). In HBNU (the institution selected for the sample in this paper), there have been no cases of COVID-19, so far. In April 2022, all teachers and students took nucleic acid tests no <3 times and no COVID-19 positive cases were identified. HBNU is, therefore, considered to be extremely COVID-19-secure. Regarding the study of human participants, the procedures used in this study are in line with the provisions of the Declaration of Helsinki. The institutional review committee approved the research protocols of the College of Physical Education of HBNU. All students voluntarily provided written informed consent before participating in the research. Participants were permitted to withdraw from the questionnaire process at any time without giving reasons. Before the survey was carried out, the theme, purpose, objectives, and scope of the study were explained to the participants, and their informed consent was obtained. The research data were kept confidential, and the identity of the participants was not disclosed.

**Table 1:** Background information of the participants.

**Elements**	***n***	**%**
**Participants**		
Students	490	100
Valid responses (after collection and using Amos)	443	90.40
**Vaccination record**		
Vaccinated SARS-CoV-2 vaccine (VeroCell)	432	97.50
Non-vaccinated	11	2.50
Received 1 vaccine dose	16	3.60
Received 2 vaccine dose	416	93.90
**Age**		
18–25 years	430	97.07
26–30 years	13	2.93
**Education**		
Undergraduate students	326	73.58
Masters students	117	26.41
**Academic major**		
Sports education	81	18.28
Social sports guidance and management	15	3.39
Physical education	39	8.80
Sports training	235	53.05
Sports humanities and sociology	44	9.93
Physical education and training	29	6.55
**Gender**		
Male	242	54.63
Female	201	45.37
**Marital status**		
Unmarried	439	99.10
Married	4	0.90

### Questionnaire Survey

The authors used a self-designed questionnaire as the survey instrument. The questionnaire was designed in the Chinese language with the help of previous data, existing literature, and the authors' own previous research experience and knowledge (25–27). Each questionnaire took approximately 10–12 min to complete. We divided the questionnaire into four main parts: 1. The background information of participants using a nominal scale; 2. Physical activity participation scenario of participants with the help of interval and ratio scales; 3. Impact of vaccination on daily life; and 4. Impact of vaccination on PA by using binary (yes/no) and ordinal scale (Likert scale ranging from 1 much better to 5 much worse) approaches (28). The survey was based on open and closed questions, such as “How often do you participate in sports on average each week?,” “Did you have a headache after vaccination?,” “Did you feel any weakness after vaccination?,” “Did you feel a loss of appetite after vaccination?,” “What is the impact of vaccination on your academic and daily life?,” “Do you think you have experienced differences in performance in terms of speed following vaccination?,” “In what other areas do you feel that performance is significantly worse after vaccination compared to before vaccination?,” and so on. It is worth noting that these questions can be found in most physical activity questionnaires, such as the International Physical Activity Questionnaire (IPAQ), although of course, such questionnaires lack the COVID-19 vaccine-related information, which was the focus of this study. The validity procedures for this survey included pilot studies, training of surveyors, and data collection quality control. We pretested the questionnaire with almost 50 students using a purposive sampling approach and modified the questions according to the findings, i.e., typing/logical errors or questions, which were found difficult to analyze statistically. In addition, we calculated Cronbach's alpha α value of all four parts to evaluate the reliability of the questionnaire. This value was 0.76, indicating acceptable internal consistency (29).

### Statistical Analysis

**Figure 2:**
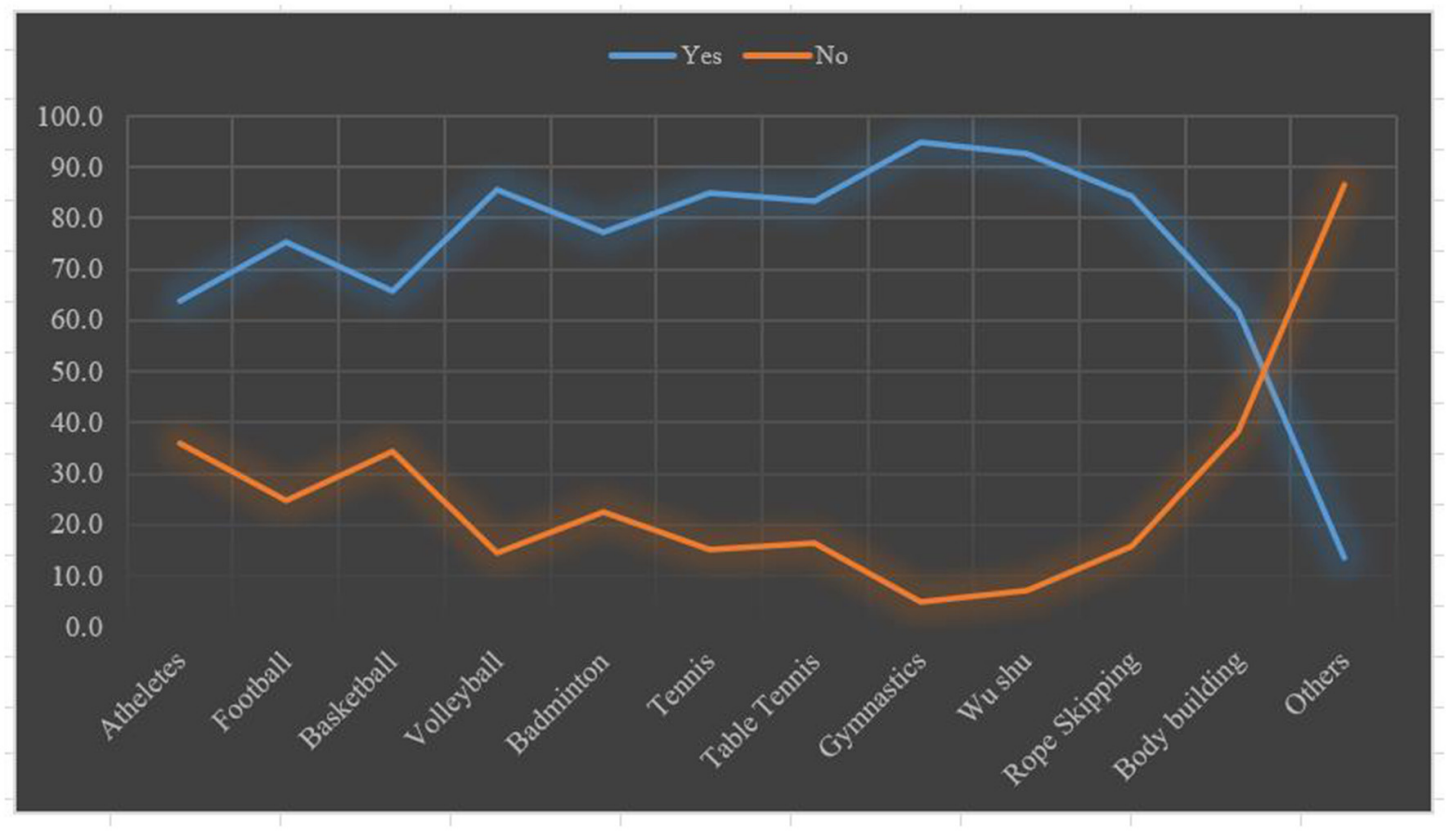
Sports in which students participated.

**Figure 3:**
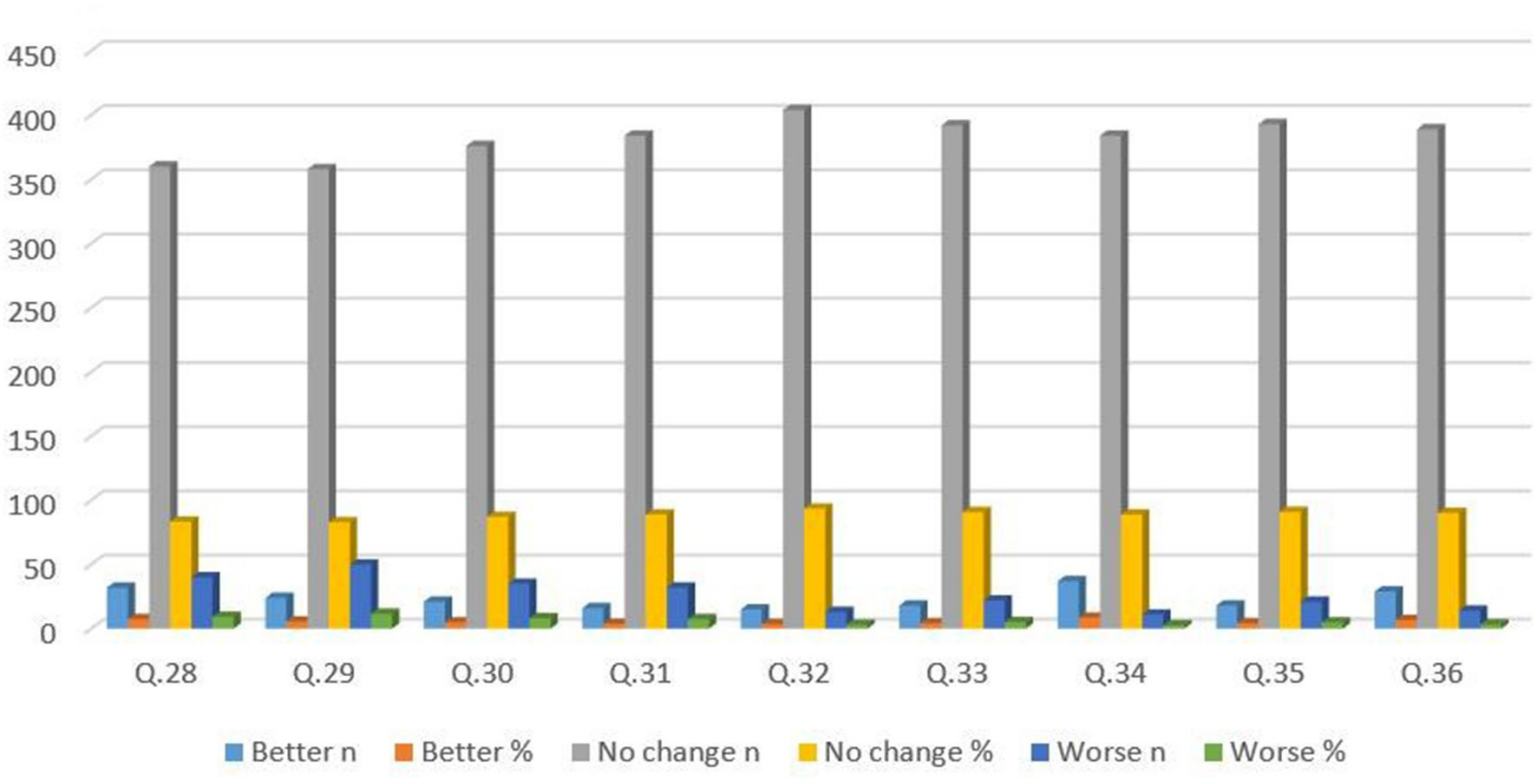
Impact of COVID-19 vaccine on physical activity performance. The percentage of the responses is according to 432 participants who were vaccinated (including students who had received only 1 vaccination). Q28. Do you think there are differences in performance in terms of speed after vaccination? Q29. What do you think are the differences in strength performance after vaccination? Q30. What do you think are the differences in endurance performance after vaccination? Q31. What do you think are the differences in flexibility performance after vaccination? Q32. What do you think are the differences in performance in terms of sensitivity after vaccination? Q33. What do you think are the differences in coordination performance after vaccination? Q34. What do you think are the differences in interest in exercise after vaccination? Q35. What do you think are the differences in participation time in sports after vaccination? Q36. What do you think are the differences in skill acquisition after vaccination?

The data were analyzed using Amos 24 and SPSS Statistics 23. We presented the background information of the participants in the shape of descriptive statistics followed by confirmatory factor analyses (CFA) and the internal consistency reliability of the instrument was measured to assure the quality of the study. Due to prior knowledge of the number of factors in the early stages of the questionnaire development (30), a one-factor model was hypothesized. That model was examined using confirmatory maximum likelihood (ML) factor analyses' parameter estimates in AMOS 24. Data were filtered by eradicating all the flawed samples. Five measures of model fit were highlighted: incremental fit index (IFI), goodness-of-fit index (GFI), comparative fit index (CFI), root mean square error of approximation (RMSEA), and difference of chi-square and degree of freedom (χ2/df) 1.3 <2. According to Hu and Bentler (31), the cut-off value of IFI, GFI, and CFI is close to 0.90, and a cut-off value close to 0.06 for RMSEA is needed to evidence a relatively good fit. However, a standard value of (χ2/df) should be <2, to authenticate the model (data). Examination of the multiple fit indices revealed that GFI, IFI, and CFI were 0.96, 0.92, and 0.99, respectively, and RMSEA was 0.06, indicating a relatively good model fit. However, χ2/df = 1.50 (Table 2). In addition, descriptive statistics, factor loadings with *h*^2^, and independent-sample *t-*test were conducted to analyze the impact of vaccines on the students' health status and PA performance (Figure 2; **Tables 3**–**5**). Lastly, the Pearson product correlation of the factors was used to evaluate the responses of participants who had received one or two vaccinations against everyday life and health impact and impact on PA performance (Figure 3; **Tables 6**–**8**).

**Table 2:** Goodness-of-fit indices of models tested.

**Model**	**GFI**	**IFI**	**CFI**	**RMSEA**	**χ2/df**
One-factor	0.96	0.92	0.99	0.059	1.50

*p = 0.05*.

## Results

After the data were collected and analyzed using Amos, 443 out of 490 (242 male and 201 female) valid responses remained (*n* = 443), showing a 90.40% response rate. Out of 443 participants, 432 were vaccinated against COVID-19. However, 16 participants had only one shot, whereas 416 participants had received two shots (see Table 1). To achieve the main purpose of this study, the authors divided the results section into four subsections. First, confirmatory factor analyses (CFA) were conducted, which provided internal consistency and reliability for the measurement of the reliability and validity of the instrument (see Table 2). Secondly, the PA situation of participants was highlighted, followed by the impact of vaccination on daily life. Finally, the PA was statistically analyzed, and the results were interpreted.

### Internal Consistency Reliability

Internal consistency concerns the interrelationship of a group of factors (32). Internal consistency estimation (alpha reliability) (33) indicated that the alpha of the scale was higher than the recommended standard of 0.70 (Alpha coefficient = 0.82). In addition, **Tables 5**, **7** show the validity and factor load of all items in both instruments with *h*^2^.

### Physical Activity Participation of Participants

The results showed that 47% of students participated in physical activity 3–4 days per week, whereas only seven (1.6%) students were not engaging in any PA at all (Table 3). Of those students who participated in PA, most of them (69.8%) exercised for 30–90 min each time, as shown in Table 3. Notably, more than 50% of students had participated in PA for 1–6 years. The standard deviation (SD) of elements indicated that the values were close to the mean (see Table 3).

**Table 3:** Physical activities of the participants.

**Elements**		***N***	**%**	**Mean ±SD**
How often do you participate in PA on average each week?	do not participate	7	1.6	3.26 0.89
	1–2 days	71	16.0	
	3–4 days	208	47.0	
	5–6 days	114	25.7	
	Everyday	43	9.7	
How long do you participate in PA on average each time?	30 min or less	15	3.4	3.03 1.03
	30–60 min	129	29.1	
	60–90 min	180	40.6	
	90–120 min	64	14.4	
	120 min or more	55	12.4	
How long have you been participating in PA?	for 1 year	57	12.9	3.11 1.29
	1–3 years	87	19.6	
	4–6 years	141	31.8	
	7–9 years	68	15.3	
	10 years and above	90	20.3	

The above Table 3 is a line chart showing the sports most played by the students. According to the results, gymnastics and Wushu (Chinese martial art) were the sports most popular with the students. Gymnastics and Wushu were practiced by 95 and 92.8% of students, respectively (Figure 2). On the other hand, athletics and body-building were in the lowest position in the list of sports, in which the students participated.

### Impact of Vaccination on Daily Life and Health

Most of the students did not experience any negative reaction, such as headache, feeling feverish, chills, or cough (94.2, 96.3, 99.5, and 98.6%, respectively) after their COVID-19 vaccination, irrespective of whether they had received one or two shots (Table 4). However, around 30–40% of the participants felt drowsiness, weakness, muscle pain, or swelling after their shot(s) of the COVID-19 vaccine (see Table 4).

**Table 4:** Impact of COVID-19 vaccine and health status.

**Elements**	***N***		**%**	
	**Yes**	**No**	**Yes**	**No**
Did you have a headache after vaccination?	25	407	5.80	94.20
Did you feel feverish after the vaccination?	16	416	3.70	96.30
Did you feel chills after vaccination?	2	430	0.50	99.50
Did you feel drowsy after the vaccination?	131	301	30.30	69.70
Did you have a cough after vaccination?	6	426	1.40	98.60
Did you have diarrhea after the vaccination?	24	408	5.60	94.40
Did you have nausea after vaccination?	5	427	1.20	98.80
Do you feel weak after vaccination?	119	313	27.50	72.50
Did you feel a loss of appetite after vaccination?	16	416	3.70	96.30
Did you feel any joint pain after the vaccination?	23	409	5.30	94.70
Did you experience any pain or swelling at the injection site after your vaccination?	176	256	40.70	59.30
Did you feel muscle pain after vaccination?	105	327	24.30	75.70
Did you experience itching at the injection site after vaccination?	39	393	9.00	91.00
Have you experienced any other uncomfortable symptoms after vaccination?	12	420	2.80	97.20
Do you think overall, that vaccination impacts your academic and daily life?	83	349	19.20	80.80

*Responses of 432 participants who had been vaccinated*.

The difference in the responses regarding the impact of the vaccine on daily life between the participants who had received one or two COVID-19 vaccination shots was analyzed. For all the elements, the responses were similar, indicating that the vaccine had no significant impact on their daily life activities or health. However (*p* < 0.05), for three items: “Did you have a headache after vaccination?,” “Did you experience any pain or swelling at the injection site after your vaccination?,” and “Have you experienced any other uncomfortable symptoms after vaccination?” (see Table 5). The validity and commonalities of all items in both instruments with *h*^2^ are highlighted in Table 5. Notably, the Pearson product correlation of participants receiving one and two shots of vaccine with impact on daily life and health were found to be highly positive to moderately positive with the *r-*value ranging from 0.50 to 0.90, respectively, and *p* < 0.05 (see Table 6). However, a few elements showed low positive correlation, i.e., Q15, Q19, and Q22, with *r*-values of 0.45, 0.46, and 0.46, respectively (see Table 6).

**Table 5:** Responses of participants receiving 1 and 2 vaccinations with factor loadings and *h*^2^.

**Elements**	**Vaccine doses**	**Mean**	**±SD**	***t*-value**	***p*-value**	**Factor loadings**	***h* ^2^**
Did you have a headache after vaccination?	1	1.81	0.4	−2.27	0.02	0.59	0.68
	2	1.95	0.22				
Did you feel feverish after the vaccination?	1	2.00	0.0	0.80	0.43	0.89	0.66
	2	1.96	0.19				
Did you feel chills after vaccination?	1	2.00	0.0	0.28	0.78	0.9	0.76
	2	2.00	0.07				
Did you feel drowsy after the vaccination?	1	1.56	0.5	−1.19	0.23	0.67	0.66
	2	1.70	0.46				
Did you have a cough after vaccination?	1	2.00	0.0	0.48	0.63	0.81	0.87
	2	1.99	0.12				
Did you have diarrhea after the vaccination?	1	2.00	0.0	0.99	0.32	0.88	0.77
	2	1.94	0.23				
Did you have nausea after vaccination?	1	2.00	0.0	0.44	0.66	0.92	0.59
	2	1.99	0.11				
Do you feel weak after vaccination?	1	1.63	0.5	−0.91	0.36	0.91	0.74
	2	1.73	0.45				
Did you feel a loss of appetite after vaccination?	1	2.00	0.0	0.80	0.43	0.69	0.54
	2	1.96	0.19				
Did you feel any joint pain after the vaccination?	1	2.00	0.0	0.97	0.33	0.85	0.71
	2	1.94	0.23				
Did you experience any pain or swelling at the injection site after your vaccination?	1	1.19	0.4	−3.40	0.00	0.79	0.65
	2	1.61	0.49				
Did you feel muscle pain after vaccination?	1	1.56	0.5	−1.85	0.06	0.71	0.64
	2	1.76	0.42				
Did you experience itching at the injection site after vaccination?	1	2.00	0.0	1.28	0.20	0.74	0.79
	2	1.91	0.29				
Have you experienced any other uncomfortable symptoms after vaccination?	1	1.81	0.4	−4.03	0.00	0.84	0.86
	2	1.98	0.15				
Do you think overall, that vaccination impacts your academic and daily life?	1	1.13	0.3	−0.37	0.71	0.91	0.95
	2	1.17	0.44				

*Participants who received 1 vaccination against COVID-19 and those who received 2 vaccinations showed no significant difference (p > 0.05 in most cases)*.

**Table 6:** Pearson correlation of participants receiving 1 and 2 vaccinations with impact health status items.

	**Q11**	**Q12**	**Q13**	**Q14**	**Q15**	**Q16**	**Q17**	**Q18**	**Q19**	**Q20**	**Q21**	**Q22**	**Q23**	**Q24**	**Q25**	**Q26**
**Q11**	1															
**Q12**	0.71**	1														
**Q13**	0.72**	0.76**	1													
**Q14**	0.83**	0.77**	0.83**	1												
**Q15**	0.45**	0.58**	0.54**	0.51**	1											
**Q16**	0.79**	0.77**	0.85**	0.94**	0.52**	1										
**Q17**	0.67**	0.83**	0.70**	0.80**	0.56**	0.82**	1									
**Q18**	0.80**	0.78**	0.88**	0.92**	0.51**	0.95**	0.79**	1								
**Q19**	0.46**	0.57**	0.55**	0.51**	0.63**	0.54**	0.58**	0.53**	1							
**Q20**	0.79**	0.73**	0.81**	0.83**	0.53**	0.87**	0.75**	0.88**	0.55**	1						
**Q21**	0.67**	0.89**	0.72**	0.79**	0.54**	0.79**	0.83**	0.80**	0.60**	0.74**	1					
**Q22**	0.46**	0.49**	0.39**	0.46**	0.32**	0.46**	0.46**	0.44**	0.46**	0.44**	0.47**	1				
**Q23**	0.50**	0.42**	0.53**	0.54**	0.38**	0.56**	0.45**	0.56**	0.54**	0.54**	0.49**	0.42**	1			
**Q24**	0.60**	0.75**	0.70**	0.70**	0.46**	0.70**	0.78**	0.71**	0.49**	0.64**	0.76**	0.37**	0.50**	1		
**Q25**	0.80**	0.79**	0.84**	0.86**	0.50**	0.82**	0.70**	0.85**	0.52**	0.76**	0.77**	0.39**	0.49**	0.62**	1	
**Q26**	0.58**	0.68**	0.61**	0.61**	0.59**	0.64**	0.63**	0.64**	0.53**	0.62**	0.63**	0.31**	0.40**	0.57**	0.59**	1

**Correlation is sinificant at the 0.01 level (2-tailed). Q11, How many vaccination doses have you received? Q12, Did you have a headache after vaccination? Q13, Did you feel feverish after the vaccination? Q14, Did you feel chills after vaccination? Q15, Did you feel drowsy after the vaccination? Q16, Did you have a cough after vaccination? Q17, Did you have diarrhea after the vaccination? Q18, Did you have nausea after vaccination? Q19, Do you feel weak after vaccination? Q20, Did you feel a loss of appetite after vaccination? Q21, Did you feel any joint pain after the vaccination? Q22, Did you experience any pain or swelling at the injection site after your vaccination? Q23, Did you feel muscle pain after vaccination? Q24, Did you experience itching at the injection site after vaccination? Q25, Have you experienced any other uncomfortable symptoms after vaccination? Q26, What is the impact of vaccination on your academic and daily life?

### Impact of Vaccination on Physical Activity Performance

Figure 3 shows the frequency and percentage of the elements related to PA performance. According to the results of this study, most participants (82–91%) expressed no change in their PA performance after receiving the COVID-19 vaccine (including participants who had received only one shot). Furthermore, 3.40–8.50% of students claimed that their performance had been better since receiving the vaccine dose(s). However, between 2.50% and maximum, 11.50% of the participants said that their PA performance had been worse since receiving the COVID-19 vaccine (Figure 3).

The authors compared the responses of the participants who had received one or two COVID-19 vaccinations. These showed similar responses for almost all the elements (indicating the performance-related scale in PA) except the element “What do you think are the differences in strength performance after vaccination?” and “What do you think are the differences in coordination performance after vaccination?” with (*p* < 0.05) (Table 7). The validity and commonalities of all items in both instruments with *h*^2^ are highlighted in Table 8. Notably, the Pearson product correlation of participants receiving one and two shots of vaccine with impact on PA performance items was found to be moderate positive with an *r-*value range from 0.50 to 0.70 with *p* < 0.05 (Table 8).

**Table 7:** Differences between participants who received 1 and 2 vaccinations with factor loadings and *h*^2^.

**Elements**	**Vaccine doses**	**Mean**	**±SD**	***t*-value**	***p*-value**	**Factor loadings**	***h* ^2^**
Do you think there are differences in performance in terms of speed after vaccination?	1	3.13	0.5	1.10	0.27	0.67	0.75
	2	2.98	0.51				
What do you think are the differences in strength performance after vaccination?	1	3.31	0.4	2.37	0.02	0.91	0.58
	2	3.04	0.46				
What do you think are the differences in endurance performance after vaccination?	1	3.00	0.0	−0.18	0.86	0.82	0.66
	2	3.02	0.42				
What do you think are the differences in flexibility performance after vaccination?	1	3.00	0.0	−0.44	0.66	0.76	0.71
	2	3.06	0.50				
What do you think are the differences in performance in terms of sensitivity after vaccination?	1	3.00	0.0	0.11	0.91	0.78	0.49
	2	2.99	0.35				
What do you think are the differences in coordination performance after vaccination?	1	2.81	0.4	−2.21	0.03	0.82	0.53
	2	3.01	0.34				
What do you think are the differences in interest in exercise after vaccination?	1	3.00	0.0	0.63	0.53	0.59	0.65
	2	2.93	0.43				
What do you think are the differences in participation time in sports after vaccination?	1	3.06	0.2	0.67	0.50	0.83	0.51
	2	3.00	0.37				
What do you think are the differences in skill acquisition after vaccination?	1	3.00	0.0	0.67	0.50	0.72	0.63
	2	2.92	0.48				

*Most of the responses of the participants who received 1 COVID-19 vaccination and those who received 2 vaccinations are not significantly different (p > 0.05 in most cases)*.

**Table 8:** Pearson correlation of participants receiving 1 and 2 vaccinations with impact on PA performance items.

	**Q11**	**Q28**	**Q29**	**Q30**	**Q31**	**Q32**	**Q33**	**Q34**	**Q35**	**Q36**
**Q11**	1									
**Q28**	0.56**	1								
**Q29**	0.58**	0.84**	1							
**Q30**	0.65**	0.74**	0.81**	1						
**Q31**	0.61**	0.70**	0.75**	0.93**	1					
**Q32**	0.69**	0.71**	0.79**	0.81**	0.76**	1				
**Q33**	0.72**	0.77**	0.84**	0.88**	0.82**	0.92**	1			
**Q34**	0.62**	0.60**	0.66**	0.71**	0.65**	0.92**	0.77**	1		
**Q35**	0.67**	0.69**	0.78**	0.82**	0.74**	0.90**	0.87**	0.87**	1	
**Q36**	0.59**	0.49**	0.57**	0.57**	0.55**	0.82**	0.63**	0.91**	0.78**	1

**Correlation is significant at the 0.01 level (2-tailed). Q11, How many vaccination doses have you received? Q28, Do you think there are differences in performance in terms of speed after vaccination? Q29, What do you think are the differences in strength performance after vaccination? Q30, What do you think are the differences in endurance performance after vaccination? Q31, What do you think are the differences in flexibility performance after vaccination? Q32, What do you think are the differences in performance in terms of sensitivity after vaccination? Q33, What do you think are the differences in coordination performance after vaccination? Q34, What do you think are the differences in interest in exercise after vaccination? Q35, What do you think are the differences in participation time in sports after vaccination? Q36, What do you think are the differences in skill acquisition after vaccination?

## Discussion

On completion of the current dataset analyses, a 90.40% submission rate was achieved concerning completed questionnaires from students located in different parts of China. The results of this study provide insight into the impact of COVID-19 vaccination on the students' daily life, health, and PA performance *via* this self-designed instrument, which passed reliability and validity tests. In this real-world study, we found that all the participants practiced up to two different sports (gymnastics and Wushu being the most popular PA among participants), and very few of the students said that they did not participate in any sports/PA, since they were pursuing academic majors within the PE department.

We observed that most students did not complain of the impact on their health status after being vaccinated against COVID-19. In addition, when we analyzed those individuals who had received one and two doses of the of COVID-19 vaccine, there was almost no significant difference between their responses regarding the impact of the COVID-19 vaccine on daily life and health. These findings are also comparable with studies such as Tande (34) and Romero (35); according to those studies, the vaccine does not have any negative impact on health and people should be encouraged to receive vaccine dose(s). Similarly, the Pearson correlation of the items (receivers of one and two shots of vaccine with impact on daily life and health items) was highly positive to moderately positive, which shows that COVID-19 vaccinations do not have a negative impact on the participants' daily life and health, and these findings fit well with the previous studies [see (34, 36)]. As far as the effects of the COVID-19 vaccine on the PA performance of the students are concerned, we observed that, overall, most of the respondents did not perceive any clear reaction or change in performance after receiving the COVID-19 vaccine. In addition, it was observed that, most of the students who received one or two shots of the COVID-19 vaccine showed very similar responses, indicating that the vaccine had no significant impact on their performance during PA. The Pearson product correlation was moderately positive. Which emphasizes that the COVID-19 vaccine had no significant negative impact on the PA performance of the students. These findings are supported by the previous literature, which indicates that there is no impact of CO the VID-19 vaccine observed in the published data (37, 38).

This study provided a platform that focused on some of the main concerns necessary to understand the physical reactions to the COVID-19 vaccine, in this case, health issues and PA performance. A self-designed questionnaire was proposed and administered to respond to the evolving impacts of the COVID-19 pandemic vaccine on daily life. The findings of this study match some previous studies, which indicated that the COVID-19 vaccine has no side effects and encouraged people to be vaccinated [see (34–36)]. In particular, according to Liu et al., (13, 14), there are high rates of vaccine hesitancy in China, which could pose a serious threat to the preventive measures aimed at controlling COVID-19 spread in the country. The results of this study also reassure us that the COVID-19 vaccine is safe. In this way, people can be encouraged to get vaccinated, which in turn can allow lives to return to normal, the world economy to recover, and also reduce the isolation, which has led many people to suffer from various NCDs (7, 39). The approach of using questionnaires has also allowed us to enhance our database by the addition of data relating to the COVID-19 vaccine. It is important to emphasize that the results presented in this study are simply a broad evaluation highlighting signs associated with COVID-19 vaccine side-effects in PE students.

The greatest strength of this study is that its results could contribute to the eradication of COVID-19 vaccine hesitancy, which in turn could eliminate isolation and help people to lead normal a life. One limitation of our study is that it did not compare male and female student responses regarding the impact of the COVID-19 vaccine. In addition, because this study is novel, its results cannot be supported by the existing literature. A further limitation is that our analysis did not compare the responses of vaccinated and non-vaccinated participants. The sample was small and, therefore, may not properly represent the whole population, and the study population was only university students that's why focused only on students. William A. Rushing, in his study, observed that rates of mental health and marital status are re lated (40). However, this study included many more unmarried than married participants. Unfortunately, the marital status ratio could not be controlled as this would have been difficult in the population of students. Neither were we able to conduct physiological experiments in laboratories to measure the parameters of the participants' body reactions before and after the vaccine. Other factors, such as cross-sectional study consequences and vaccination effect, may also cause variations in results under different circumstances.

Future work requires global research on the COVID-19 vaccine. This cooperative effort can promote the development, treatment, and prevention of COVID-19, promote the development of communities and medical support systems and promote evidence-based policy decisions. By encouraging people to receive COVID-19 vaccinations, meaningful assistance and prevention measures can be generated. These measures may benefit vulnerable groups with a poor quality of life in society.

## Conclusion

The impact of the COVID-19 vaccine on daily living activities, health, and sports performance was evaluated and reported in detail. The purpose of the study was achieved by the effective use of a self-designed questionnaire. By taking the example of university students' current study is trying to overcome vaccine hesitancy in the general population not only limited to China. According to the results of this study, it is concluded that the COVID-19 vaccine had no significant effect (or only a very minor effect) on the participants' daily living activities, health status, and PA performance. These results are consistent with previously published studies showing no impact of the COVID-19 vaccine on participants (34–36). Although more data on COVID-19 are still being gathered, the current findings highlight the effects of the COVID-19 vaccine targeted population and the related health consequences.

## Data Availability Statement

The raw data supporting the conclusions of this article will be made available by the authors, without undue reservation.

## Ethics Statement

The studies involving human participants were reviewed and approved by Hubei Normal University. The patients/participants provided their written informed consent to participate in this study.

## Author Contributions

RAL: conceptualization. RAL and ZZ: methodology. RM and RAL: writing—original draft preparation. LZ and SZ: writing—review and editing. XF, WW, and SL: validation. All authors have read and agreed to the published version of the manuscript.

## Conflict of Interest

The authors declare that the research was conducted in the absence of any commercial or financial relationships that could be construed as a potential conflict of interest.

## Publisher's Note

All claims expressed in this article are solely those of the authors and do not necessarily represent those of their affiliated organizations, or those of the publisher, the editors and the reviewers. Any product that may be evaluated in this article, or claim that may be made by its manufacturer, is not guaranteed or endorsed by the publisher.

## Abbreviations

PA: physical activity
COVID-19: coronavirus disease of 2019
PE: physical education.
